# Modulation of spatiotemporal dynamics in the bromate–sulfite–ferrocyanide reaction system by visible light

**DOI:** 10.1039/d2ra01422j

**Published:** 2022-05-18

**Authors:** Mengfei Liu, Chunxiao Meng, Ling Yuan

**Affiliations:** College of Chemical Engineering, China University of Mining and Technology Xuzhou 221116 China yuanling_1984@163.com

## Abstract

We have carried out the first systematic study of the effects of visible light on the homogenous dynamics in the bromate–sulfite–ferrocyanide (BSF) reaction. Under flow conditions, the reaction system displayed photoinduction and photoinhibition behavior, and the oscillatory period decreased with the increase of light intensity, which is due to the fact that light irradiation mainly enhanced the negative process and affected the positive feedback. The light effect on positive and negative feedback is studied by analyzing the period length of pH increasing and decreasing in detail. With the increase of light intensity, the period length of pH increasing decreases monotonically, while the period length of pH decreasing changes nonmonotonically. These results suggest that light could be used as a powerful tool to control homogenous dynamics. Results obtained from numerical simulations are in good agreement with experimental data.

## Abstract 2

The BSF reaction system displayed photoinduction and photoinhibition behavior under flow conditions. The oscillatory period decreased as the light irradiation mainly enhanced the negative process and affected the positive feedback.

## Graphical abstract

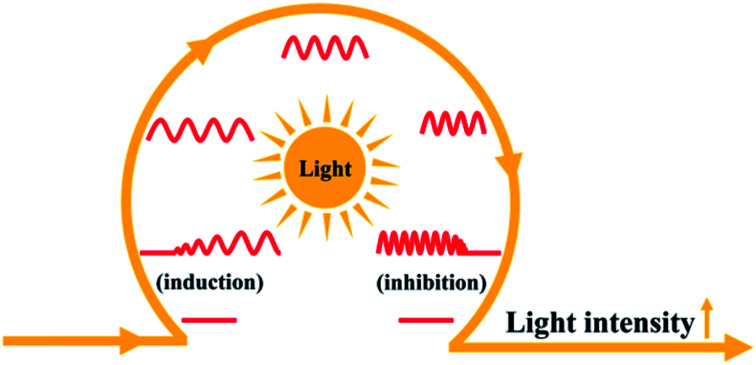

## 1. Introduction

Photo-sensitive chemical reactions play important roles in many physiological processes,^1^*e.g.*, photosynthesis,^2^ circadian clock,^3,4^ bioluminescence,^5,6^ and phototaxis.^7,8^ In addition, light as an external control parameter is particularly appealing in the study of nonlinear chemical reactions due to the easy tunability of intensity and spectral composition.^9–11^

Many chemical oscillators are known to be sensitive to light irradiation, showing photoinduction and photoinhibition behavior in homogeneous and inhomogeneous systems. In the Ru(bpy)_3_^2+^-catalyzed Belousov–Zhabotinsky (BZ) oscillator, the oscillatory frequency was influenced by the illumination intensity,^12^ while the rotating spiral waves transform to a labyrinthine standing-wave pattern by periodic light-perturbation.^13^ By virtue of the process, photophobic and phototropic movements of a self-oscillating gel hosting the photosensitive BZ reaction were designed to approach its favorable environments spontaneously.^14,15^ In another case, the photosensitive dissociation of molecular iodine was utilized in the chlorine dioxide-iodine-malonic acid (CDIMA) reaction, Briggs–Rauscher reaction, and Bray–Liebhafsky reaction, leading to the cessation or promotion of oscillations,^16–18^ and pattern structures modulation^11,19,20^ under the forcing of light illumination.

In addition, light effects on oscillatory dynamics have recently been studied in other pH chemical oscillations, especially the hydrogen autocatalytic processes.^21–26^ Photo-induced oscillations and pulse waves have been studied in the hydrogen peroxide–sulfite–ferrocyanide (HPSF) reaction.^27^ The number of oscillatory-peaks was conveniently controlled by changing the illumination period and duration. Original of the phenomena was accounted for the reversible photolysis of ferricyanide, which was followed by the protonation of newly produced cyanide. In the HPSF reaction, the negative feedbacks of the oscillation contained two loops (the consumption of hydrogen ions by ferrocyanide^22^ and the autocatalytic formation of hydroxide radical),^26^ both of which were promoted under the light illumination. Hence, the oscillation frequency was influenced by multiple factors and was not easy to control.

The bromate–sulfite–ferrocyanide (BSF) reaction also shows excellent photosensitivity under visible light. Moreover, the negative feedback of the BSF reaction is mainly the oxidation of ferrocyanide by bromate, which consumes considerable hydrogen ions produced in the positive feedback. Kaminaga and coworkers have proposed that light illumination can be used to directly tune the dynamics of the BSF reaction through accelerating the negative feedback (BrO_3_^−^–Fe(CN)_6_^4−^) and have no effect on the positive feedback (BrO_3_^−^–SO_3_^2−^).^23^ However, in the combined system (BrO_3_^−^–SO_3_^2−^–Fe(CN)_6_^4−^), the enhancement of negative feedback must indirectly affect the positive feedback process. Therefore, in this work, we systematically investigated the light effect on the frequency of oscillations in the homogenous medium of BSF reaction system by experimental and numerical methods for revealing the light effect on both negative and positive feedback.

## 2. Experimental content

### 2.1 Material

Analytical-grade reagent KBrO_3_, Na_2_SO_3_, H_2_SO_4_ and K_4_[Fe(CN)_6_]·3H_2_O (Sinopharm Chemical Regents) were used without purification. Throughout the experiment, the four reaction solutions were prepared immediately before each run by the ultrapure water supplied by a water purification system (Millipore, Milli-Q Jr) and were continuously bubbled by N_2_ in order to avoid the effect of air oxidation. It has been known to all that Fe(CN)_6_^4−^ are affected by light. For this reason, Fe(CN)_6_^4−^ aqueous solution was stored in the brown bottle wrapped up in shading cloth. In addition, all experiments were done in the dark box to avoid the influence of daylight.

### 2.2 Method

#### 2.2.1 Experimental section

The flow reactor with a total volume of 13.0 mL was made of quartz glass allowing the light to pass more easily. The following input feed concentrations were kept fixed in all CSTR experiments: [BrO_3_^−^]_0_ = 35 mM, [SO_3_^2−^]_0_ = 25 mM, [Fe(CN)_6_^4−^]_0_ = 15 mM, [H_2_SO_4_]_0_ = 4.2 mM. Four stock solutions were separately introduced into the reactor by a four-channel peristaltic pump (ISMATIC, Switzerland) through glass capillary tubes.

To avoid local acidification, the SO_3_^2−^ and H_2_SO_4_ tubes were premixed before their entering into the reactor. The reactor mixture was vigorously stirred by a Teflon-coated magnetic stirrer bar. The thermostat (PolyScience, USA) was used to maintain the reaction temperature of *T* = 30.0 °C in the reactor during the experiment. The reactor was equipped with a combined pH compound electrode (Cole Parmer, USA) to monitor the pH value. The pH-time data was recorded by a computer through a pH meter and an E-coder (Edaq 201, Australia).

An LED light source (*λ* = 385 ± 10 nm) was used as an accessory light source for controlling the homogenous dynamics by modulating the light intensity with a digital control unit and calibrating with a photometer (Model 1 L1400A, International Light). The optical path length was 2 cm.

#### 2.2.2 Simulation section

The simulations of homogenous kinetics were carried out with a commercial software package (Berkeley Madonna, error control parameter set at 10^−10^) for stiff differential equations. The same results were obtained with the error control parameter set at 10^−6^ and 10^−12^ and with another stiff algorithm.

## 3. Results and discussion

### 3.1 Effect of irradiation intensity on the oscillatory dynamics of BSF reaction systems in a CSTR

In the BSF reaction system, Fe(CN)_6_^4−^ acts as a negative feedback agent, which mainly exists in the form of protonation (HFe(CN)_6_^3−^) through (R1).^22^ In the dark, the negative feedback loop consumes six protons combined with (R1) and (R2). Under the light irradiation, the more reactive Fe(CN)_5_(H_2_O)^3−^ is formed by the key photosensitive step (R3). Meanwhile, the photo-oxidation process (R5) gives rise to the hydrated electron if the aqueous solution of Fe(CN)_6_^4−^ is exposed in UV light.^22^ In the present work, the aqueous solution of Fe(CN)_6_^4−^ was is strictly shielded from light to avoid (R5) before entering to the CSTR. Actually, the photo-oxidation process increases with decreasing excitation wavelength, as for excitation wavelength below 315 nm, the primary process is photo-oxidation with a secondary aquation process.^28,29^ Reinhard *et al.* also reported that the main product at the excitation wavelength of 266 nm are the products of the photo-oxidation process, while at 355 nm are the product of the photo-aquation process.^30^ Apart from the influence of wavelength, pH also affects the yield of photo-oxidation, the photo-oxidation process is more important on the base condition than on the acid condition.^31^ The wavelength of the light source in our experiment is 385 ± 10 nm and the pH range is between 4 and 7, which is an acid environment. Therefore, we propose that the photo-aquation process plays a dominant role in our experiment.R1
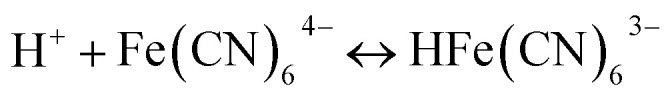
R2
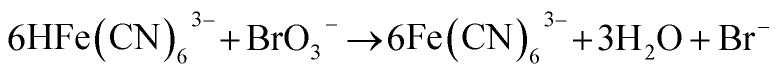
R3
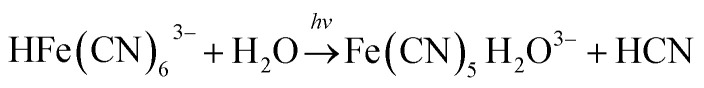
R4
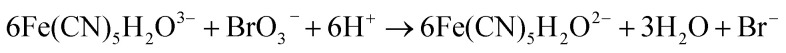
R5
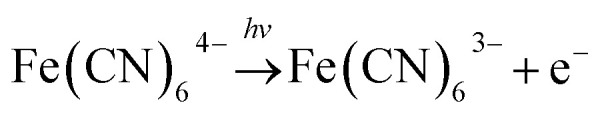


**Fig. 1:**
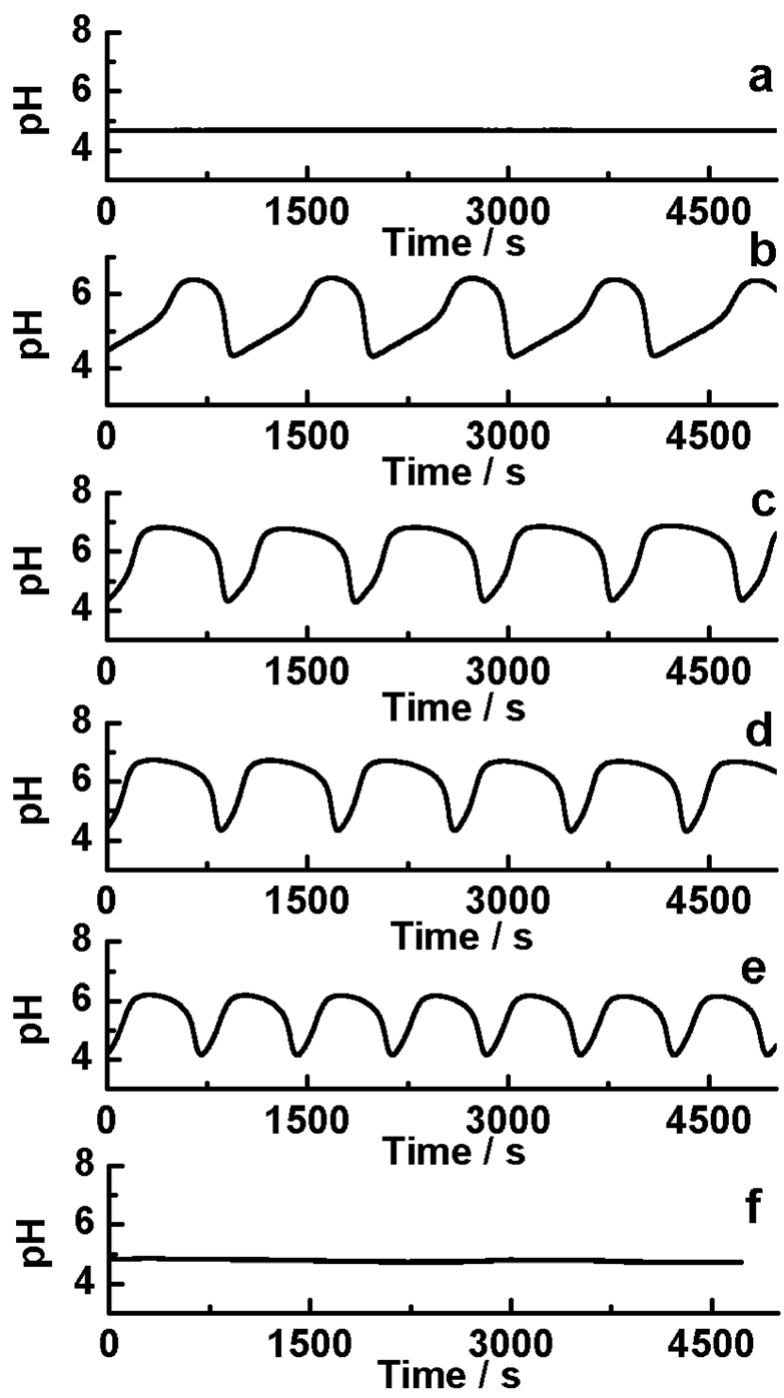
The kinetic curves under different light intensities in a CSTR. *k*_0_ = 2.78 × 10^−3^ s^−1^; *I* = (a) 0 mW cm^−2^; (b) 1.154 mW cm^−2^; (c) 3.56 mW cm^−2^; (d) 11.24 mW cm^−2^; (e) 26.9 mW cm^−2^; (f) 29.3 mW cm^−2^.

Fig. 1 shows the photo-sensitive dynamics in the CSTR. Under dark, the system shows a steady low pH state which illustrated that autocatalytic reaction was dominant (Fig. 1a). When the system was illuminated with the light, the low pH state immediately entered into an oscillatory state if irradiation intensity increased to 1.154 mW cm^−2^ (Fig. 1b). As the increasing of the irradiation intensity, the shape of the oscillatory waveform varied which mainly resulted from enhancing the negative feedback process by (R4). As the oscillatory amplitude ranged from 4 to 7, Fe(CN)_6_^4−^ mainly exists in the protonation form, so the photooxidation process was inhibited, while the main photo-response reaction was the photo-aquation HFe(CN)_6_^3−^. However, when light is applied at *I* = 29.3 mW cm^−2^, the reaction system displayed photoinhibition and reached to low pH steady-state (Fig. 1f).

**Fig. 2:**
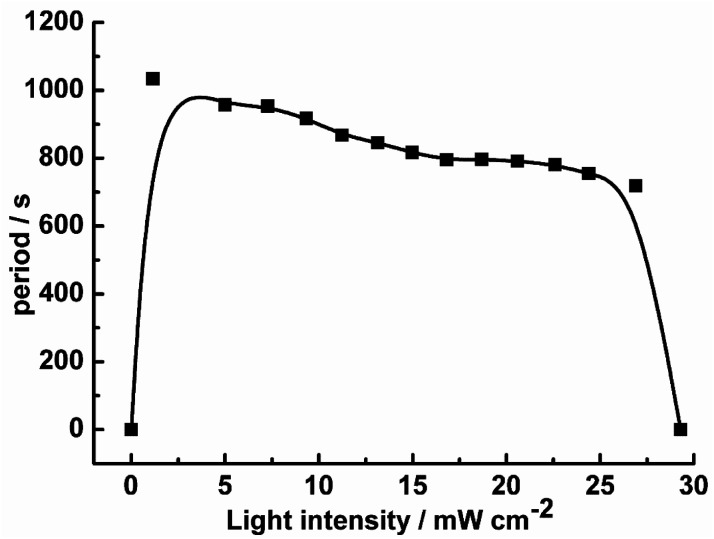
The oscillatory period as the function of light intensity in the CSTR at *k*_0_ = 2.78 × 10^−3^ s^−1^.

**Fig. 3:**
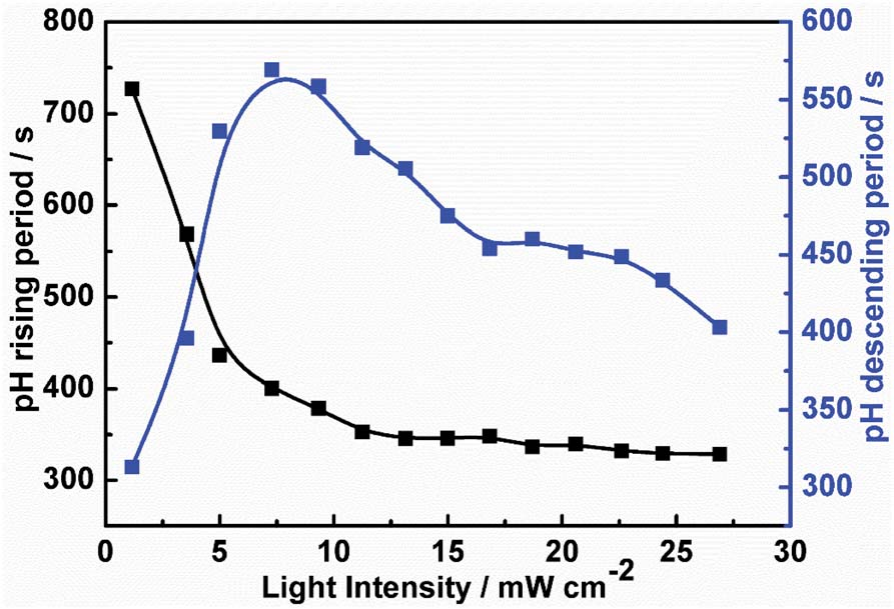
Measured pH rising period and descending period as a function of light intensity in a CSTR.

To establish the relationship between the light intensity and the oscillatory period, we have measured the periods under different light intensities, and the results show in Fig. 2. With increasing light intensity, the whole oscillatory periods change monotonically until the system goes into a steady state. The period length of the pH increasing and decreasing are also analyzed shown in Fig. 3. The pH increasing period represents the time required for the negative feedback process, while pH decreasing represents the time required for the positive feedback process. Fig. 3 shows the influence of light intensity on the positive and negative feedback process of the reaction system. As the increasing of *I*, the pH rising period decreased firstly when *I* ≤ 10.26 mW cm^−2^. At *I* > 10.26 mW cm^−2^, there is no obvious effect on the pH rising period. On the contrary, the pH descending length changed nonmonotonically with the increase of light intensity. In other words, when *I* ≤ 10.26 mW cm^−2^, visible light irradiation not only affected the negative feedback but also modulated the positive feedback. This is easy to understand, under this range of light intensity, due to the production of more reactive Fe(CN)_5_H_2_O^3−^, the negative feedback loop increased gradually, which resulted in the weaken of positive feedback. However, when *I* > 10.26 mW cm^−2^, the pH descending period changed slowly as the increasing the light intensity, this is probably because much more HFe(CN)_6_^3−^ covert to Fe(CN)_5_H_2_O^3−^ which produced more HCN and dissociated proton by (R6), resulting in strengthening positive feedback processes. So, the oscillatory period decreased as the increase of the light intensity when *I* > 10.26 mW cm^−2^.

R6 HCN ↔ CN^−^ + H^+^

### 3.2 Computational results

For explaining the experimental results in the CSTR, the mechanism of BSF reaction is given in Table 1. In the dark, the BSF reaction mechanism can be described by (M1)–(M7) reactions which has been successfully applied to simulate the homogenous dynamics of the BSF reaction.^23,32–35^ With light illumination, the composition reactions (M8)–(M10) could be considered. In addition, the reaction of Fe(CN)_6_^3−^(M11) could be considered, which also lead to the decreasing of pH. The rate laws and rate constants used in the simulations are given in Table 2. During simulation, the rate constants of (M1), (M2), (M4), (M5) was obtained from ref. 32. And others were fitted according to our experimental results.

**Table 1:** Mechanism for the photosensitive BSF reaction

No.	Reactions
M1	SO_3_^2−^ + H^+^ ↔ HSO_3_^−^
M2	HSO_3_^−^ + H^+^ ↔ H_2_SO_3_
M3	3HSO_3_^−^ + BrO_3_^−^ → 3SO_4_^2−^ + Br^−^ + 3H^+^
M4	3H_2_SO_3_ + BrO_3_^−^ → 3SO_4_^2−^ + Br^−^ + 6H^+^
M5	6H_2_SO_3_ + BrO_3_^−^ → 3S_2_O_6_^2−^ + Br^−^ + 6H^+^ + 3H_2_O
M6	H^+^ + Fe(CN)_6_^4−^ ↔ HFe(CN)_6_^3−^
M7	6HFe(CN)_6_^3−^ + BrO_3_^−^ → 6Fe(CN)_6_^3−^ + 3H_2_O + Br^−^
M8	CN^−^ + H^+^ ↔ HCN
M9	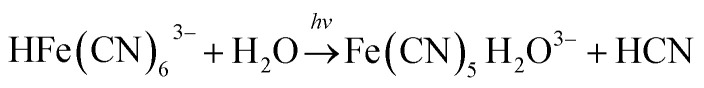
M10	6Fe(CN)_5_H_2_O^3−^ + BrO_3_^−^ + 6H^+^ → 6Fe(CN)_5_H_2_O^2−^ + 3H_2_O + Br^−^
M11	SO_3_^2−^ + 2Fe(CN)_6_^3−^ + H_2_O → 2Fe(CN)_6_^4−^ + SO_4_^2−^ + 2H^+^

**Table 2:** Rate laws and constants for the photosensitive BSF reaction

Rate law	Rate constants
*v*_1_ = *k*_1_[SO_3_^2−^][H^+^] − *k*_−1_[HSO_3_^−^]	*k*_1_ = 10^10^ M^−1^ s^−1^, *k*_−1_ = 10^3^ s^−1^
*v*_2_ = *k*_2_[HSO_3_^−^][H^+^] − *k*_−2_[H_2_SO_3_]	*k*_2_ = 6 × 10^9^ M^−1^ s^−1^, *k*_−2_ = 10^8^ s^−1^
*v*_3_ = *k*_3_[BrO_3_^−^][HSO_3_^−^]	*k*_3_ = 0.13 M^−1^ s^−1^
*v*_4_ = *k*_4_[BrO_3_^−^][H_2_SO_3_]	*k*_4_ = 18 M^−1^ s^−1^
*v*_5_ = *k*_5_[BrO_3_^−^][H_2_SO_3_]	*k*_5_ = 0.7 M^−1^ s^−1^
*v*_6_ = *k*_6_[Fe(CN)_6_^4−^][H^+^] − *k*_−6_[HFe(CN)_6_^3−^]	*k*_6_ = 10^10^ M^−1^ s^−1^, *k*_−6_ = 6 × 10^10^ s^−1^
*v*_7_ = *k*_7_[HFe(CN)_6_^3−^][BrO_3_^−^]	*k*_7_ = 0.085 M^−1^ s^−1^
*v*_8_ = *k*_8_[CN^−^][H^+^] − *k*_−8_[HCN]	*k*_8_ = 10^10^ M^−1^ s^−1^, *k*_−8_ = 6 × 10^10^ s^−1^
*v*_9_ = *αk*_9_[HFe(CN)_6_^3−^]	*k*_9_ = 0−100 s^−1^
*v*_10_ = *k*_10_[BrO_3_^−^][Fe(CN)_5_H_2_O^3−^][H^+^]	*k*_10_ = 2 M^−2^ s^−1^
*v*_11_ = *k*_11_[Fe(CN)_6_^3−^][SO_3_^2−^]	*k*_11_ = 2 M^−1^ s^−1^

The change of chemical species in time in the CSTR can be described by the following eqn (E1):E1
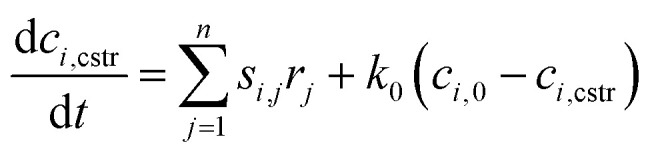
here *c*_*i*,cstr_, *c*_*i*,0_ are the concentrations of *i*th chemical species in the CSTR. *s*_*i*,*j*_ is the stoichiometric number of the *i*th species in the *j*th reaction step, *r*_*i*_ is the reaction rate of the *j*th reaction, and *n* is the number of reaction steps, *k*_0_ is the flow rate in the reactor.

During the simulation process, we define the photokinetic rate constant *K*_9_, which is proportional to the light intensity *I* (*K*_9_ = *k*_9_*I*). The photokinetic factor *α* = (1 − exp(−2.3*A*))/*A* involves the absorption of light by all the components of the system, where *A* is the total absorbance at the irradiation wavelength of 385 nm, defined as 
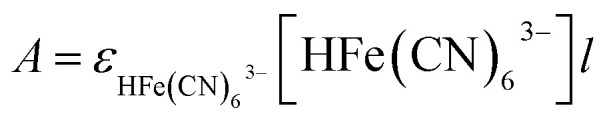
, where *ε* is the molar extinction coefficients and *l* is the optical path length in the CSTR, and 
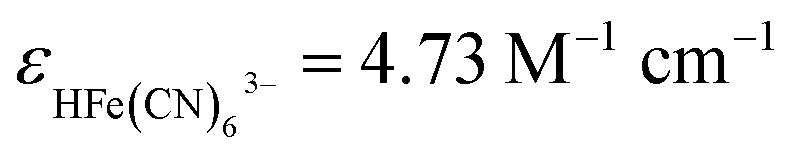
 is the molar extinction coefficients of HFe(CN)_6_^3−^, *l* is the optimal length of the reactor.

**Fig. 4:**
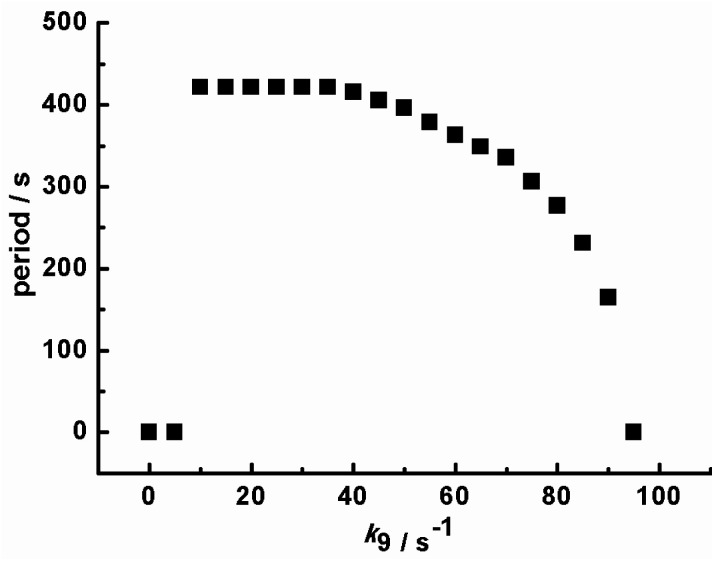
Calculated oscillation period with the increasing of photokinetic rate *k*_9_ in a CSTR. Input concentrations are the same as Fig. 2.

**Fig. 5:**
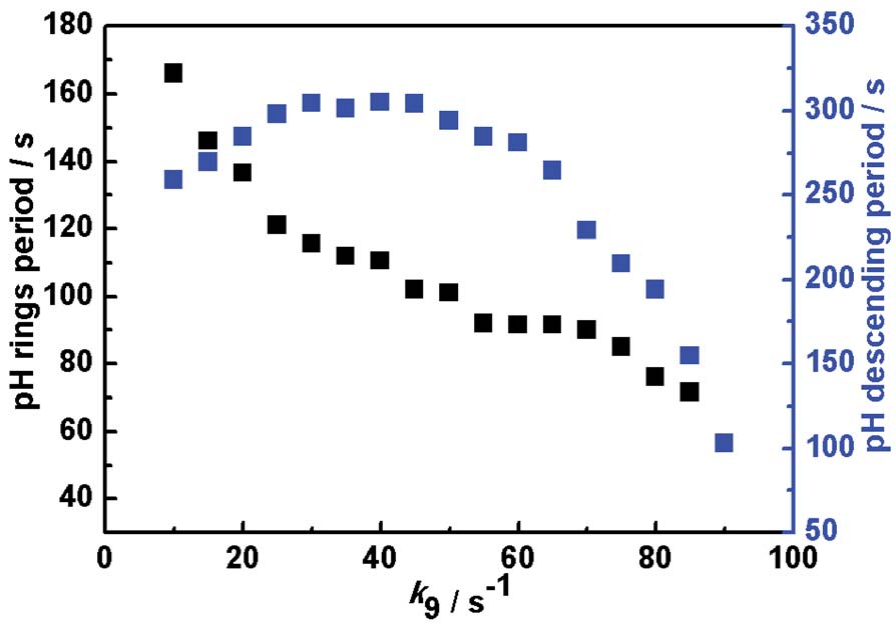
Calculated pH rising period and descending period as a function of photokinetic rate *k*_9_ in a CSTR. Input concentrations are the same as Fig. 3.

Based on the above mechanism in Table 1, the relationship between the oscillatory periods and photokinetic rate *k*_9_ in the CSTR was obtained, as shown in Fig. 4. If *k*_9_ equals zero, the system has no oscillations. As increasing *k*_9_ to 10 s^−1^, sustained pH oscillations started. The oscillatory periods gradually decreased with a further increase of *k*_9_. When the *k*_9_ is greater than 90 s^−1^, the oscillation is suppressed. The pH rising period and descending period under different *k*_9_ were also calculated, as shown in Fig. 5. The variation trend of oscillatory period obtained by simulation was consistent with the experimental results. The monotonic change of pH rising period and non-monotonic change of pH descending period obtained by simulation were also consistent with the experimental results. However, the oscillation period of the simulation results is inconsistent with the experiment, because the rate constants of many reactions cannot be obtained. Our next work will further study photoreaction kinetic of the sub-reaction system for detecting the relevant kinetic parameters.

## 4. Conclusion

To summarize, in this work we observed the light effect on the oscillations in the bromate–sulfite–ferrocyanide reaction system, which provides that light can be used as a convenient control parameter to regulate the reaction kinetics. As the increasing of light intensity, the negative feedback was enhanced, while the positive feedback was modulated. The rate law model involved eleven elemental reactions that can simulate the homogenous phenomenon well with the experimental results. The relationship between the oscillatory dynamics and illumination intensity could be obtained to facilitate further multiple application of the BSF, such as driven for self-assembly of nanoparticles,^36^ switching the conformation of a DNA molecule,^37^ and aggregation of supra-amphiphile molecules.^38,39^

## Conflicts of interest

There are no conflicts to declare.

## Note added after first publication

This article replaces the version published on 18^th^ May 2022, which contained an error in (R6).

## Supplementary Material
